# Multiple genome viewer (MGV): a new tool for visualization and comparison of multiple annotated genomes

**DOI:** 10.1007/s00335-021-09904-1

**Published:** 2021-08-27

**Authors:** Joel E. Richardson, Richard M. Baldarelli, Carol J. Bult

**Affiliations:** The Jackson Laboratory, Bar Harbor, ME USA

## Abstract

The assembled and annotated genomes for 16 inbred mouse strains (Lilue et al., Nat Genet 50:1574–1583, 2018) and two wild-derived strains (CAROLI/EiJ and PAHARI/EiJ) (Thybert et al., Genome Res 28:448–459, 2018) are valuable resources for mouse genetics and comparative genomics. We developed the multiple genome viewer (MGV; http://www.informatics.jax.org/mgv) to support visualization, exploration, and comparison of genome annotations within and across these genomes. MGV displays chromosomal regions of user-selected genomes as horizontal tracks. Equivalent features across the genome tracks are highlighted using vertical ‘swim lane’ connectors. Navigation across the genomes is synchronized as a researcher uses the scroll and zoom functions. Researchers can generate custom sets of genes and other genome features to be displayed in MGV by entering genome coordinates, function, phenotype, disease, and/or pathway terms. MGV was developed to be genome agnostic and can be used to display homologous features across genomes of different organisms.

## Introduction

The availability of multiple annotated mouse genome assemblies serves as a foundation for investigating the genomic and genetic basis for phenotype diversity across inbred and wild-derived strains of laboratory mice (Keane et al. 2011; Lilue et al. 2018; Thybert et al. 2018). However, even with the availability of widely used genome browsers, interactive and customizable comparison of annotated genome features and their organization across multiple genomes remains a significant barrier to accessing and exploring these genomes. Here we describe the multiple genome viewer (MGV) which we developed to support interactive exploration and visualization of genome annotations for multiple lines of laboratory mice and for easy access to the sequence data for these annotations for alignment and data analysis. MGV relies on the mouse genome assemblies available from the Ensembl genome browser. The genome feature annotations displayed in MGV come from the mouse genome database (MGD) unified gene catalog (Zhu et al. 2015). The MGD unified gene catalog combines genome feature predictions from Ensembl/GenCode and NCBI into a single non-redundant annotation set which serves as the foundation for the expertly curated phenotype, function, and developmental expression annotations for mouse genes available from MGD and the gene expression database (GXD) (Baldarelli et al. 2021; Blake et al. 2020; Zhu et al. 2015) Although the implementation for MGV reported here is focused on mouse genomes, the software is genome agnostic and was designed to accommodate multiple annotated genomes from any organism and can also support the display of homologous genome features across different organisms.

**Fig. 1:**
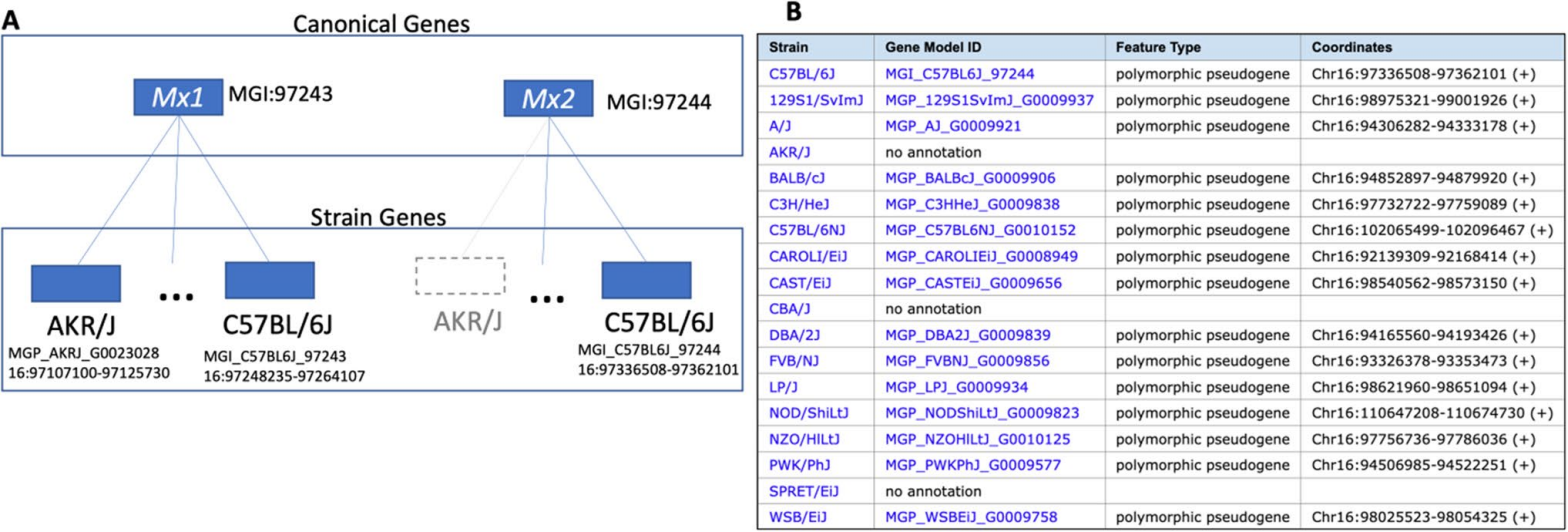
Canonical genes are the abstract representations of any/all identified mouse genome features regardless of strain. **A** Canonical genes have MGI accession ids and official nomenclature and historically have been the entities associated with biological annotations about function, phenotype, and disease. With the advent of multiple mouse genome assemblies, the concept of a strain gene was added to support robust representation of strain-specific genome annotations and attributes. Strain genes have strain-specific accession identifiers and genome coordinates. A given canonical mouse gene may or may not exist in a specific mouse strain, as shown for *Mx2* (MGI:97244), which has not been annotated in the AKR/J inbred strain. **B** The strain distribution table for the *Mx2* gene showing the strain-specific identifiers and genome locations. This table is available from the gene detail page in MGD. The *Mx2* gene is a polymorphic pseudogene meaning the gene codes for a functional protein in some strains, but not others

To represent genome features of multiple strains, MGD uses the concept of a canonical genome feature (Fig. 1A). A canonical genome feature is one that exists in any strain or species of *Mus*. In MGD, any genome feature that has a unique, permanent MGI accession id is a canonical feature. Canonical gene records are linked to instances of the genome feature found in the genome of different *Mus* strains and species. The canonical genome feature concept supports rapid identification of strain-specific genome features and the capacity to associate biological annotations with specific strains. MGD provides a tabular summary of the strain distribution of genome features along with their strain-specific identifiers and genome coordinates (Fig. 1B).

## Results

### MGV’s user interface

**Fig. 2:**
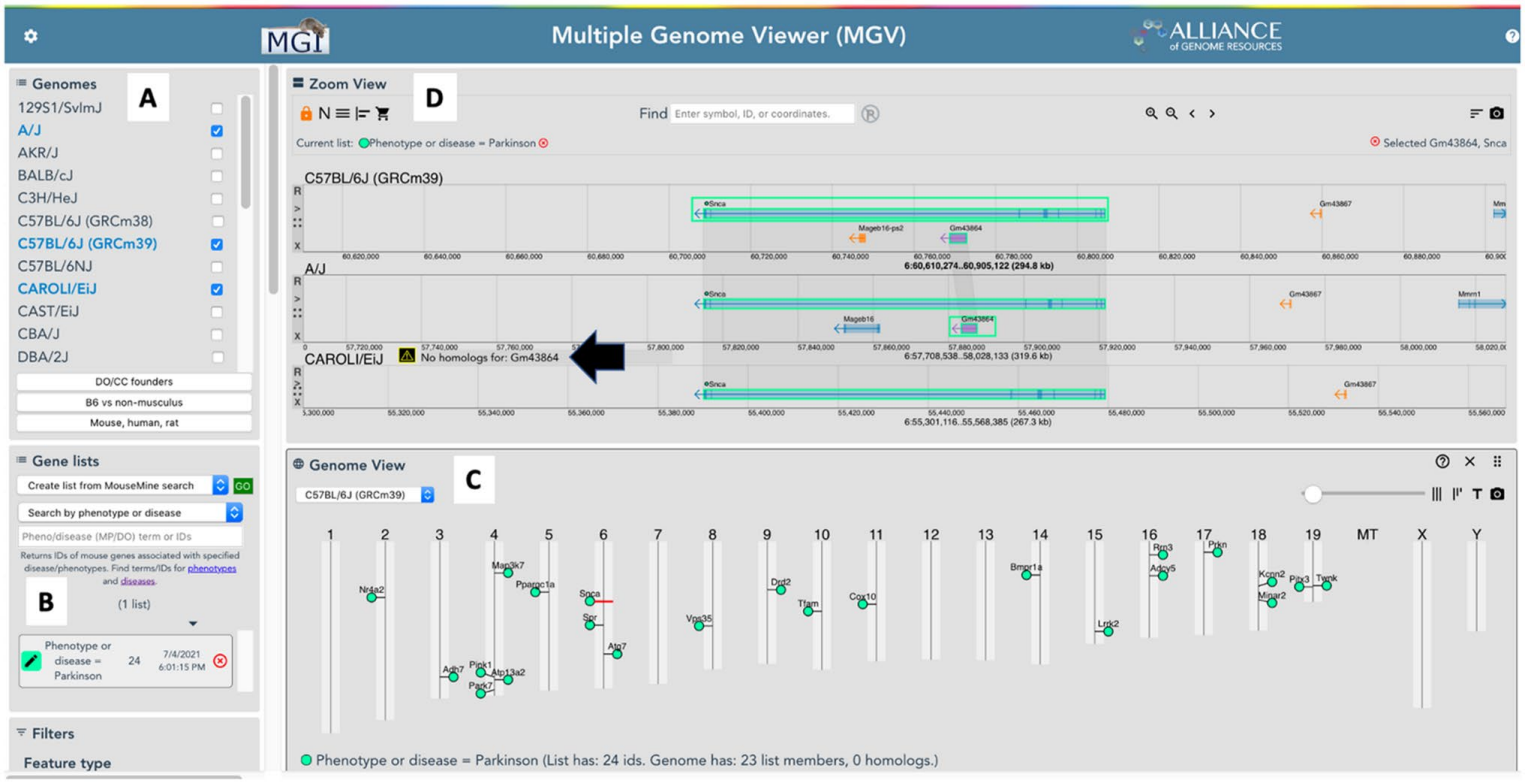
Screenshot of the MGV user interface showing results of a Gene List search for mouse genes associated with Parkinson’s disease. Note: not all display function modules are shown in this figure. **A** The genomes module allows users to select which genome tracks to display. Here, C57BL/6J, A/J, and CAROLI/EiJ have been selected. **B** Users can search for sets of mouse genes by various attributes. Here, the search was for genes in the mouse genome database (MGD) annotated to the disease term, Parkinson’s disease. **C** The results of the gene set search for Parkinson’s disease are automatically displayed on the genome map. **D** Swim lane connectors highlight equivalent features across the genome tracks. Here, the *Snca* gene (MGI:1277151) is highlighted showing its location across the three user-selected genomes. Gene, *Gm43864* (MGI:5690256), is only annotated to the C57BL/6J and A/J genomes. A message is displayed on the CAROLI/EiJ track to indicate that *Gm43864* is not found in that genome (black arrow)

The user interface for MGV consists of a control panel, tracks for displaying genome feature maps of user-selected genomes, and a genome-wide overview (Fig. 2). The control panel on the left side of the display comprises several modules that allow users to control which genomes and features to display, options to modify display properties, controls for downloading sequences, and the ability to create and display lists of features. The basic functions for MGV are described below. The MGV user guide lists the key and mouse driven commands for the interface and can be invoked at any time while using the software with the H command.

#### User interaction

The user interacts with MGV with both the mouse and with keyboard commands. For example, dragging on a displayed region scrolls the region, while pressing the ‘H’ key opens or closes the Help screen. Many commands have both mouse- and key-based versions, e.g., the left and right arrow keys can also be used to scroll. MGV uses key combinations to modify commands. For example, holding down the shift key while dragging (shift-drag) zooms into the specified region. Note that the “option” key is “alt” on some keyboards; likewise, the “command” key is sometimes “meta”. The Help screen lists all the mouse and key commands.

#### Genome selection

Any number of genomes can be selected for display from a list of those available. For the laboratory mouse, preselected groups of genomes– including the eight founder strains of the Collaborative Cross (Iraqi et al. 2008) are also available. In addition to comparing annotations across different mouse genomes, MGV can be used to compare the annotations of different assembly versions of the same genome. For example, MGV includes annotated genomes for both GRCm38 and the most recent GRCm39 assemblies of the C57BL/6J reference genome. To support comparisons of orthologous genome features across different organisms, MGV includes annotated reference genome assemblies of human (*Homo sapiens*), fruit fly (*Drosophila melanogaster*), zebrafish (*Danio rerio*), nematode (*Caenorhabditi elegans*), rat (*Rattus norvegicus*), and yeast (*Saccharomyces cerevisiae*). Annotations for these genomes and the definitions of orthologous genome features are obtained from the Alliance of Genome Resources (Alliance of Genome Resources 2019; Alliance of Genome Resources Consortium 2019).

By design, MGV has no predefined reference genome; any genome can be selected as the reference, which then determines which regions are shown for other displayed genomes. Selecting a reference genome is optional; however, other modes support aligning the genomes around common landmarks, or even using each displayed genome as an independently scrollable and zoomable browser.

#### Feature selection

Genome features are selected by clicking on them. Multiple features can be selected by pressing the shift key while clicking. Selected features are highlighted and may show other differences depending on other control settings. For example, one can use the contrast slider (under settings) to fade unselected features, or the expand setting to show the transcripts of selected features (or of all features).

#### Display filters

The genome features displayed in MGV can be toggled on and off according to multiple criteria, including feature biotype (i.e., protein coding, pseudogene, etc.), feature length, features present in some displayed genomes but not others, and features that have been selected by the user.

#### Display controls

MGV provides several tools for modifying display attributes, including font size for feature labels, feature thickness, gap width between transcripts, and feature highlight properties. These controls allow the user to highlight specific genes while deemphasizing others and produce more compact views in regions of high density. Combined with the image download function, they are also useful for producing images for publication.

#### Linking and bookmarking

MGV supports many parameters in the URL used to link to it, and it continually updates the browser’s address bar to reflect the user’s current view. At any point, the user can bookmark the view or copy/paste the URL into a link. As well, the browser’s back/forward buttons work to undo/redo navigation changes.

#### Gene lists

MGV supports a lightweight notion of lists, which are simply collections of canonical feature IDs. Features in a list can be highlighted in the display, the display can be limited to just the features in a list, or list features can be removed from the display. Lists can be created by entering IDs, by selecting features in the display, and by querying biological annotations such as disease and phenotype associations. For example, a researcher can create a list of mouse genes associated with Parkinson’s disease based on expertly curated information from MGD, display those genes on a genome overview map, and then select one or more genes from the genome map to explore in detail across strains.

#### Sequence download

A powerful feature of MGV is the ability to select and download sequences from displayed genomes. Selecting sequences adds descriptors to a sequence cart which functions like an online shopping cart. From the sequence cart, researchers can download the sequences for their selected genome features in FASTA format to a file, browser tab, or to the clipboard. Because the sequence cart contains only descriptors, there is no limit to the number of sequence features listed there. However, a single download operation is limited to a maximum of 100 MB and 4000 sequences.

To select genomic, transcript or CDS (coding sequence) sequences for a specific gene (and optionally for all its homologs) a user can open the gene’s context menu (right click on the gene) and choose the appropriate option. By default, CDS sequences are added with amino acid translation, but this setting can be disabled in the Sequence Cart. MGV users can also select sequences for genomic regions of interest by option-dragging with the mouse. If the scroll lock (lock icon in control area) is on, the same regions are selected from all the displayed genomes; otherwise, the selection applies only to the specific genome. The direction that the user drags the mouse determines whether reverse complementation is initially active (right to left) or inactive (left to right).

#### Image download

The user can download an image of the current view by clicking the camera icon in the main navigation panel. By default, the image is downloaded in PNG (portable network graphics) format. Shift-clicking the camera icon downloads the image in SVG (scalable vector graphics) format. Compared to a direct screen capture, the image download function returns the entire view, including sections that may be scrolled out of view. As well, the SVG option allows the image to be rendered at any desired resolution. User control over font size, track spacing, and other display parameters also aid in producing images for publication.

### Use cases

The primary driver for the development of MGV was to allow researchers to visualize and explore genome features and their organization across different mouse genomes. The use cases described below are examples for two of the main uses of MGV: exploring annotation similarities and differences across the genomes of multiple mouse strains and comparing the organization of homologous genome features from different organisms.

#### Display and explore genome-specific annotation differences

The ease with which sequences (genomic, cDNA, CDS, reverse complement, etc.) can be accessed with MGV greatly simplifies the comparative genome annotation analysis needed to identify annotations that are in one genome and not another and to determine if the condition represents true strain-specific differences or is the result of incomplete genome assemblies and/or missing annotations (Lilue et al. 2018). The MGV display align feature provides a comparative view of a given genome feature to identify similarities and differences between genomes for that feature. While not a multiple sequence alignment tool, the MGV display alignment functionality centers the view on a selected feature, aligns corresponding features at their 5′ end across the genome tracks for all the user-selected genomes, and dynamically scales each genome so the selected feature occupies approximately the same span. The sequences that underlie the genome features are easily accessed and saved using MGV’s sequence cart and subsequently submitted to external sequence alignment and analysis tools.

**Fig. 3:**
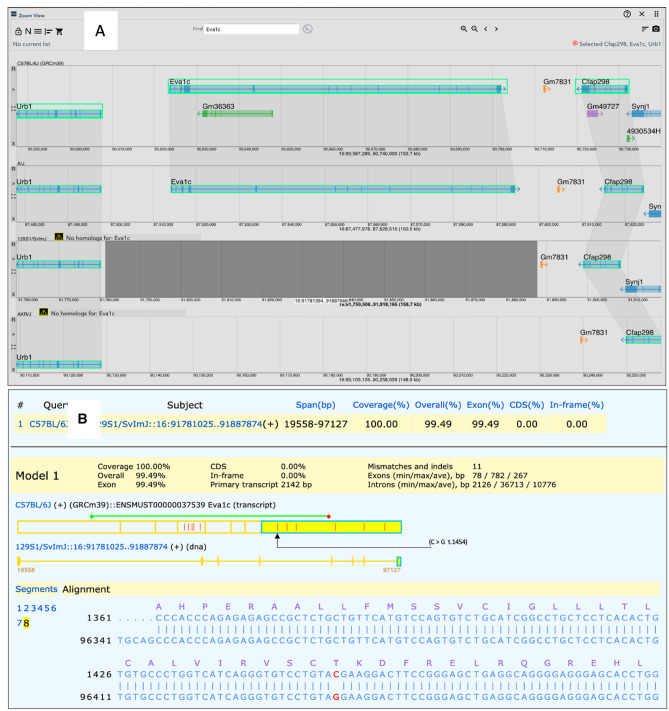
MGV can aid in the evaluation of missing annotations in different mouse strains. **A** A region of Chr16 is shown flanked by the *Urb1* and *Cfap298* genes (MGI:2146468 and MGI:1915251, respectively). Vertical connections (swim lanes) between features indicate equivalent annotations in those genomes. The *Eva1c* gene is annotated in several mouse strains (C57BL/6J, and A/J shown), but not in others (129S1/SvImJ, AKR/J shown). **B** NCBI Splign tool output using sequences from MGV’s Sequence Cart function showing that *Eva1c* is present in the 129S1/SvImJ genome, but is not annotated due to incomplete annotation of the genome assembly. The 5′ end of the 8th cDNA/genomic alignment is shown, with conceptual translation. Differences between aligned sequences are indicated by red vertical bars in the transcript segments. All sequence variants in the CDS are synonymous, except for a C > G (B6 > 129) non-synonymous transversion (Thr > Arg) at position 1454 in the transcript (black arrow)

For example, aligning the display of annotations across multiple strains for the *Eva1c* gene (MGI:1918217) shows that this gene has been annotated in several mouse strains, but not in 129S1/SvImJ and other strains (Fig. 3A). The expression of *Eva1c* (a novel Slit receptor gene) is expressed in axons of developing forebrain and spinal cord and likely plays a role in Slit/Robo mediated axon guidance (James et al. 2013). We used MGV’s sequence extraction functions in conjunction with the National Center for Biotechnology Information (NCBI) Splign tool (Kapustin et al. 2008) to further investigate the putatively missing *Eva1c* annotations. The Splign tool efficiently compares cDNA to genomic sequence, provides useful displays of sequence alignments that highlight differences, and overlays CDS translations on the alignments. We used MGV’s Sequence Cart function to download genomic sequence from strains that either lacked or included the *Eva1c* annotation and then performed an alignment with an *Eva1c* transcript sequence from C57BL/6J (ENSMUST00000037539) using Splign. The alignments revealed that the *Eva1c* gene is, in fact, present in the 129S1/SvImJ genome (Fig. 3B). We also investigated coding sequence variation in *Eva1c* between C57BL/6J and the strains with missing *Eva1c* annotations. The majority of the CDS region variation between the C57BL/6J *Eva1c* transcript and the genomic sequences for 129S1/SvImJ are synonymous substitutions. Similar results were observed for other strains with apparent missing *Eva1c* annotation (AKR/J, CAST/EiJ, CBA/J, DBA/2J, LP/J, PWK/PhJ and SPRETUS/EiJ; data not shown). The number of SNPs observed between the C57BL/6J *Eva1c* sequence and strains missing the *Eva1c* annotation is higher on average than between strains with the annotation (8–25 SNPs for strains without the *Eva1c* annotation compared to 0–7 SNPs for strains with the annotation). For exons contained in the C57BL/6J *Eva1c* transcript used, in no strain was a nonsense substitution observed and only a single conserved indel was observed in the 5′-UTR for two strains that lack the *Eva1c* annotation (DBA/2J and PWK/PhJ). As expected, *Eva1c* annotations from wild-derived strains, CAROLI/EiJ and PAHARI/EiJ, show more variation when compared to C57BL/6J *Eva1c* (46 SNPs/4 UTR indels, 99 SNPs/6 UTR indels, respectively). That none of the variation observed in any of these strains interrupts the *Eva1c* CDS region strongly supports the suggestion that this gene is functional in these strains and that the missing *Eva1c* gene is a matter of incomplete annotation.

**Fig. 4:**
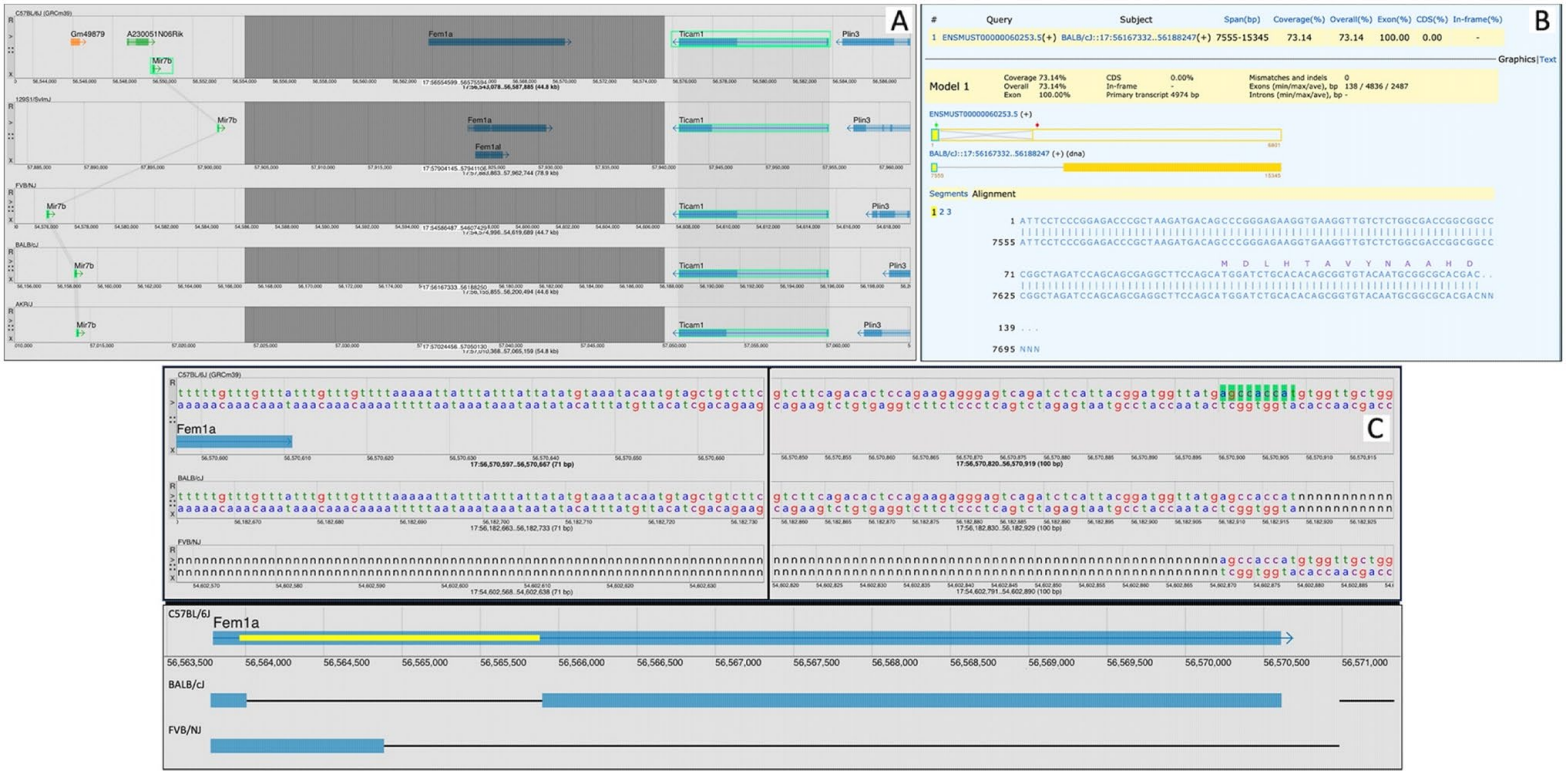
MGV can reveal genome issues with genome assemblies. **A** The *Fem1a* region on Chr17 is shown for genomes of strains C57BL/6J, 129S1/SvImJ, FVB/NJ, BALB/cJ, and AKR/J. The region shown is bounded by the *Ticam1* and Mir7b genes (highlighted) to illustrate that the *Fem1a* region is bounded by common annotations in all strains. *Fem1a* annotations are present in C57BL/6J and 129S1/SvImJ, but absent from the other strains shown. **B** NCBI Splign alignment of C57BL/6J Fem1a transcript ENSMUST00000060253 against the BALB/cJ genomic sequence flanked by *Ticam1* and *Mir7b*. **C** *Fem1a* region gaps in the BALB/cJ and FVB/NJ genomes on Chr17. Top panel: base pair-level resolution in the MGV for a region at the 3′ end of the Fem1a gene in C57BL/6J, BALB/cJ, and FVB/NJ. The three genomes were manually aligned on a 9 bp sequence that borders opposing gaps in BALB/cJ and FVB/NJ (green highlight). Vertical black bar represents ~ 182 bp excluded for display. Bottom panel: schematic overview of sequence gaps in the Fem1a gene region of BALB/cJ and FVB/NJ strain genomes. Full C57BL/6J Fem1a transcribed region shown with GRCm39 coordinates and CDS (yellow bar). Relative locations of sequence gaps in BALB/cJ and FVB/NJ show as thin black lines

A second case study for comparative genome annotation assessment using MGV centers on the *Fem1a* gene (MGI:1335089) on chromosome 17 (Fig. 4). The *Fem1a* gene encodes an ankyrin repeat-containing protein that interacts with prostaglandin E2 receptor 4 (*Ptger4;* MGI:104311) to inhibit NF-κB-mediated inflammation in macrophages (Minami et al. 2008). We investigated the missing *Fem1a* annotations on chromosome 17 in several mouse strains using the same approach for *Eva1c* described above using MGV’s sequence cart function to see if these strain differences were real and potentially biologically significant or due to technical issues. The analysis revealed that the missing *Fem1a* annotations in several strains are not due to strain divergence but are instead linked to sequence gaps in the current genome assemblies on chromosome 17 (Fig. 4B,C).

While most of the available mouse genomes have the *Fem1a* gene located on chromosome 17, this gene is currently annotated on chromosome 11 in strains BALB/cJ and FVB/NJ. Most strains have a *Fem1a*-related gene (*Fem1al*; MGI: 2441689) annotated on chromosome 11, but for several strains (129S1/SvImJ, C57BL/6NJ, LP/J, WSB/EiJ) the *Fem1al* gene is annotated to chromosome 17 (data not shown). The sequence gaps in the genome assemblies identified using the MGV on chromosome 17 will need to be resolved before the relationships between *Fem1a*-related sequences on chromosomes 11 and 17 across different mouse strains can be fully and accurately characterized.

**Fig. 5:**
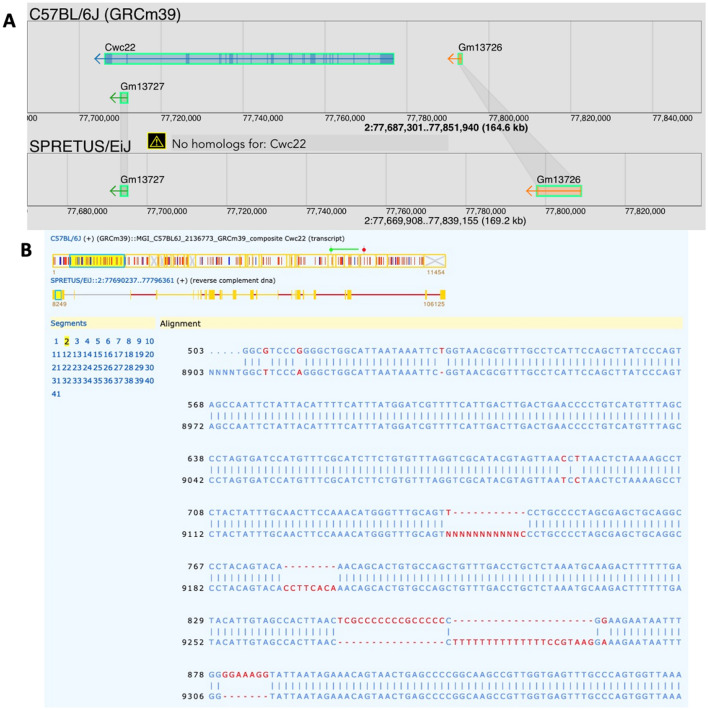
**A** MGV aligned view of the *Cwc22* gene showing the missing annotation in Spretus/EiJ. **B** NCBI Splign alignment of the *Cwc22* transcript from C57BL/6J to the Spretus/EiJ genome reveals a remnant of the *Cwc22* gene in Spretus with severe evolutionary decay in the sequence

The strain distribution of the *Cwc22* gene (CWC22 spliceosome-associated protein; MGI:2136773) illustrates a difference in annotation across mouse strains that is the result of evolutionary divergence as opposed to technical issues with genome sequence quality and completeness. *Cwc22* is annotated in all of the available mouse strain genomes with the exception of the Spretus/EiJ genome (Fig. 5). The gene is contained within the R2d segmental duplication region of the mouse genome (Morgan et al. 2016). We aligned a transcript of the *Cwc22* gene from C57BL/6J to the Spretus/EiJ genome using Splign and revealed the existence of a remnant of the gene in Spretus with significant sequence decay (Fig. 5). Future updates of genome features in the Spretus/EiJ genome should include the annotation of a *Cwc22* pseudogene.

#### Display and explore genome annotations across organisms

**Fig. 6:**
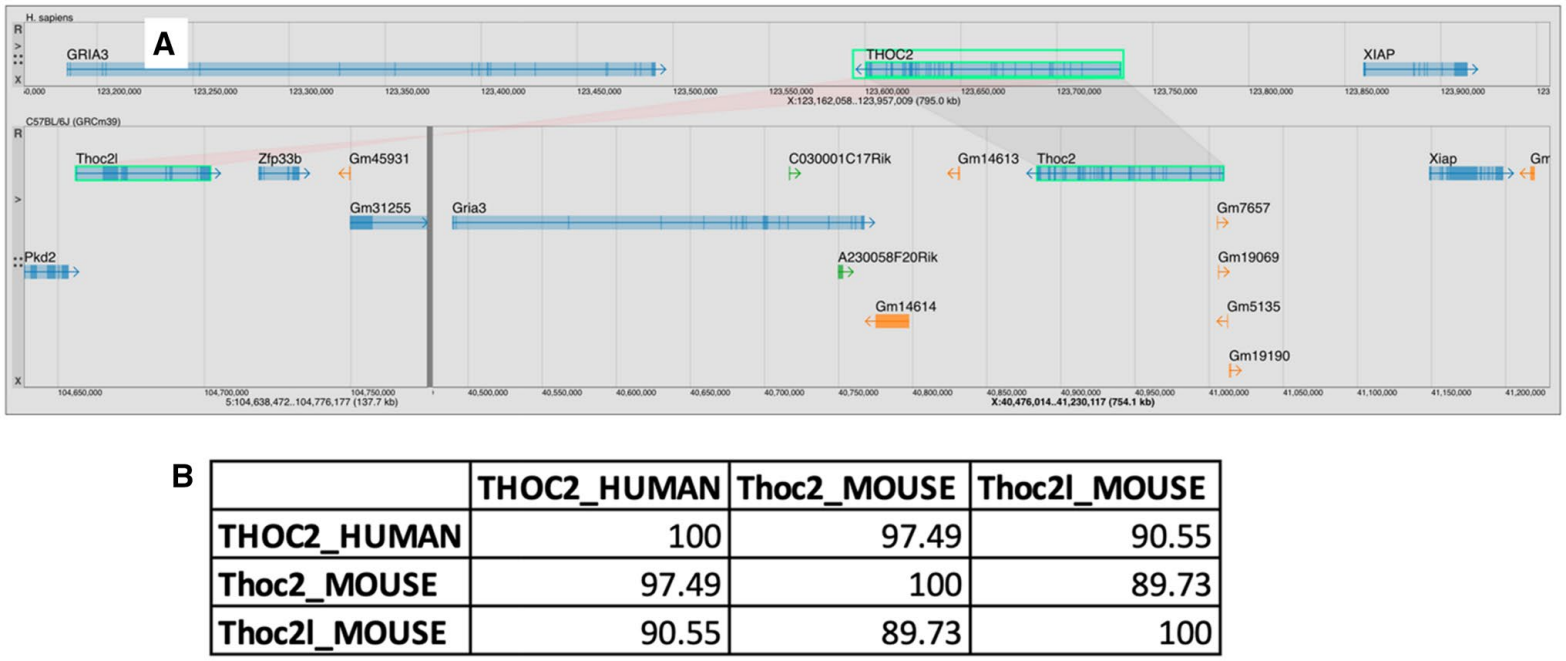
**A** MGV aligned view of the human *THOC2* gene on human Chr X and its two mouse orthologs, *Thoc2* and *Thoc2l*, on mouse Chr X and Chr 5, respectively. MGV automatically splits the view to show all orthologs of the selected feature in the compared genomes (dark vertical line in the C57BL/6J genome). Relative transcriptional orientation between orthologs is indicated by the color of the swim lanes (light gray or light red for same and opposite orientations, respectively). **B** Amino acid percent identity matrix between human THOC2 (NP_001075019), mouse THOC2 (ENSMUSP00000044677), and mouse THOC2L (ENSMUSP00000130629), produced using Clustal Omega showing that the protein produced by the *THOC2L* paralog has greater sequence similarity to human THOC2 than to mouse *THOC2*

MGV supports the exploration of the genome context of orthologous genes from different organisms. For example, there are two mouse orthologs of the human *THOC2* gene (THO Complex 2; HGNC:19073): *Thoc2* (MGI:2442413) and *Thoc2l* (MGI:3040669) located on chromosomes X and 5, respectively (Fig. 6). The human *THOC2* gene is associated with the disease X-linked intellectual disability-short stature-overweight syndrome (DOID:0112056; OMIM #300957). Aligning the MGV display on human *THOC2* produces a useful split view of the two mouse orthologs (Fig. 6). MGV does this automatically for all orthologs of a selected feature in the genomes displayed where orthologs in a given comparison genome are present on different chromosomes or located outside of the rendered view. It is clear from the genome context that the mouse chromosome X locus is in a region of conserved synteny with respect to human *THOC* (Fig. 6A).

An amino acid percent identity matrix generated by downloading protein sequences to MGV’s sequence cart and sending them to Clustal Omega (Sievers et al. 2011) reveals that the mouse THOC2 protein is 97.5% identical to the human protein (Fig. 6B). The mouse THOC2L protein is also highly similar to human THOC2 (90.6% amino acid identity) (Fig. 6B). In fact, the mouse THOC2L protein is more similar to human THOC2 than it is to mouse THOC2. The design of genome editing reagents to generate mouse models of X-linked intellectual disability-short stature-overweight syndrome should consider the high sequence similarity of mouse *Thoc2l* gene on chromosome 5. While the *THOC2* case described above is a relatively simple example, MGV is designed to easily visualize and interact with multiple orthologs at one time. As each genome section containing an ortholog of the selected feature has independent browsing operations, exploration of genome context in each segment and generation of customized views are possible.

**Fig. 7:**
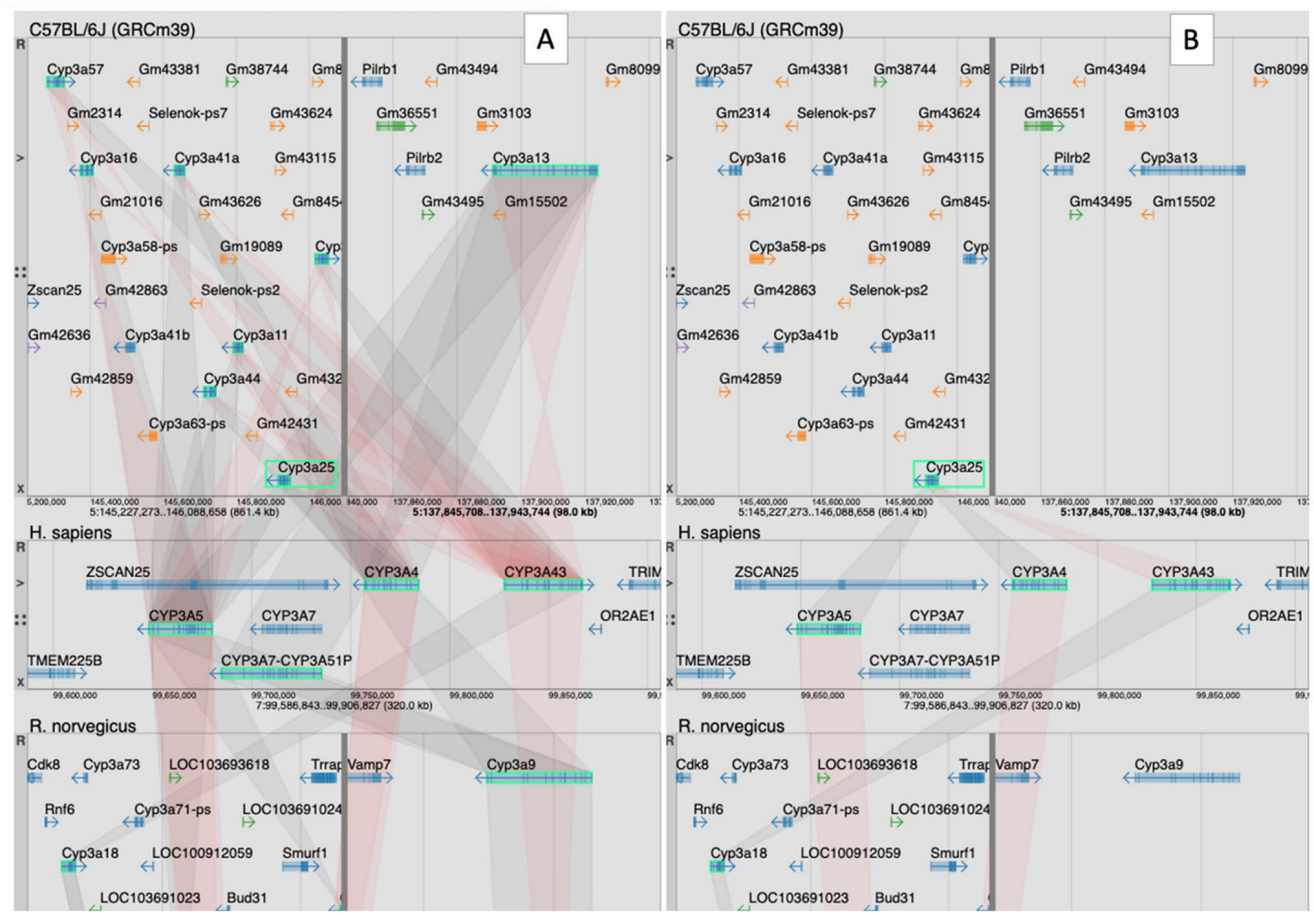
Screenshot of MGV showing mouse *Cyp3a25* in C57BL/6J, human, and rat with (**A**) and without (**B**) inferred paralogs displayed. MGV uses data from the Alliance of Genome Resources (https://alliancegenome.org) for non-mouse gene models and orthology assertions. The color of the swim lane connecting orthologs indicate if the orientation of the genome feature is in the same (light gray) or opposite (light red) orientation. Relationships of genome features across organisms can be complex due to biology and/or genome annotation and assembly issues

Because MGV supports dynamic scaling of multiple genome regions and has numerous display control options, it is very well suited for comparative analyses of multi-gene families (Fig. 7). For example, the cytochrome P450 family of monooxygenases are central to the metabolism of endogenous compounds and also for drug metabolism. The CYP3A subfamily catalyzes the largest fraction of the many P450-mediated metabolic clearance reactions known (Rendic and Guengerich 2015). Mouse models are important in predicting efficacy and potential human side effects of novel therapeutics, but effective model depiction must consider differences in the P450 gene repertoires between human and mouse (Henderson et al. 2019). MGV provides homology-based views of annotated gene family members based on strict definitions of orthologs from the Alliance of Genome Resources (Alliance of Genome Resources 2019; Alliance of Genome Resources Consortium 2019). An MGV view of relationships between the mouse *Cyp3a25* gene (MGI:1930638) and orthologs in human and rat is shown in Fig. 7B. Human orthologs of mouse *Cyp3a25* include the *CYP3A4* (HGNC: 2637) and *CYP3A5* (HGNC: 2638) genes, known to account for over 30% of the metabolic reactions involving marketed and developmental therapeutic drugs (Rendic and Guengerich 2015). MGV allows users to toggle between strict orthologous connections between gene family members and a view of inferred paralogous relationships (Fig. 7A). The paralog option provides a view of the complexity of this gene family in the mouse genome, and MGV provides easy access to sequence data from each family member for further analysis to aid in the design of humanized mouse models in those cases where multiple mouse genes need to be replaced with their human gene equivalents (Henderson et al. 2019).

## Methods

### Implementation and availability

MGV is accessible from the mouse genome informatics (MGI) web site (http://www.informatics.jax.org/mgv/). By default, the viewer provides access to genome features for 19 mouse genomes and annotations from the reference genomes for human (*H. sapiens*), fruit fly (*D. melanogaster*), zebrafish (*D. rerio*), nematode (*C. elegans*), rat (*R. norvegicus*), and yeast (*S. cerevisiae*). For the mouse reference genome, the annotations for the GRCm38 (mm10) and GRCm39 (mm11) assemblies are available.

MGV comprises two software components: the viewer itself, a JavaScript application running in the browser, and the scripts (Python and shell) for preparing the data files used by the viewer. Both software components are available at GitHub: https://github.com/mgijax/mgv and https://github.com/mgijax/mgv_data, respectively.

The viewer is a single-page JavaScript web app written with the Vue.js reactive framework. A reactive framework (angular and react are two other examples) simplifies web applications by providing features such as template-based rendering and component definition/composition combined with reactivity, wherein the rendered scene automatically updates to reflect changes in the data. These features are used throughout the viewer code, from organizing the page structure down to rendering features in a genomic region.

Genomic regions are rendered as SVG (scalable vector graphics), a widely used standard language for drawing 2D scenes on the Web. From a programming standpoint, SVG offers several attractive features, especially combined with templates and reactivity. Drawing a genomic region becomes more declarative, and the application’s code can focus on data manipulation. The main issue with using SVG is rendering performance for large scenes when there are many thousands of nodes. MGV provides ways for the user to adjust the amount of rendered detail but in general, performance degradation may be noticeable for large regions and/or many genomes.

The mgv_data repository provides the scripts to build the “back end” which serves data to the viewer at runtime. By default, these scripts are configured to build the same data sets being served by the MGV instance deployed at MGI, i.e., the genome assemblies and gene model annotations for nineteen inbred strains of mice and six non-mouse organisms. Customizing the build for other organisms would be relatively straightforward, especially for those available at Ensembl or NCBI. At runtime, the back end comprises many static files (GFF3, plain text, and JSON) and one Python CGI script. Most data requests from MGV are for the static files, while sequence retrievals are served by the CGI.

### Summary and future directions

As more complete genomes become available for the laboratory mouse and other species of mouse, the ability to compare genome features and genome organization across different individuals and strains will become increasingly important for investigations into how genome differences relate to differences in biological function. MGV is a versatile and extendable software tool with powerful interactive features to support comparative genomics research. The ability for researchers to highlight genome features across different genomes is one of the features of the software that sets it apart from other available genome browsers. As illustrated in the examples provided in this report, MGV supports visualization and analysis functions that support the evaluation of annotation differences across strains to determine if they are true differences or are due to technical issues with the current genome assemblies.

Several features are under development for future releases of MGV. One of the major enhancements planned for MGV is support for comparisons of the structure of individual genes (e.g., introns, exons, transposable elements, endogenous retroviral insertions sites, etc.) across different strains of mice. As described in Keane et al. (Keane et al. 2011), differences in gene structure across mouse strains have been documented for many genes including, *Prdm9*, *Mcm9*, and *Poli*. Allowing researchers to quickly compare gene structures will be important for identifying putative functional variants in the genomes of different mouse strains and species. Future versions of MGV will also support upload of private annotated genomes and the integration and visualization of gene expression and any other data type that can be represented meaningfully using standard genome annotation and display formats such as GFF, WIG, and BED (http://genome.ucsc.edu/FAQ/FAQformat). The current inferences of paralogs used in MGV will be replaced with paralog assertions provided by the Alliance of Genome Resources when these become available. We will also extend the gene list functionality of MGV to include annotations for model organisms other than the laboratory mouse by integrating The Alliance of Genome Resources recently released AllianceMine database as the annotation source. Finally, we will add the new rat genome assembly (mRatBN7.2; https://tbmkl.xyz/5b531c0a1a4d134a5513100e441e4f0c) to support comparing annotations on this recently released assembly to the previous one for rat (Rnor_6.0).

## Data Availability

MGV is accessible from the mouse genome informatics (MGI) web site (http://www.informatics.jax.org/mgv/).

## Code availability

The software for the MGV viewer and the scripts for preparing the data files used by the viewer are available at GitHub: https://github.com/mgijax/mgv and https://github.com/mgijax/mgv_data, respectively.
